# Preclinical development of a long-acting trivalent bispecific nanobody targeting IL-5 for the treatment of eosinophilic asthma

**DOI:** 10.1186/s12931-022-02240-1

**Published:** 2022-11-19

**Authors:** Linlin Ma, Min Zhu, Guanghui Li, Junwei Gai, Yanfei Li, Huaiyu Gu, Peng Qiao, Xiaofei Li, Weiwei Ji, Rui Zhao, Yue Wu, Yakun Wan

**Affiliations:** 1 School of Medical Technology, Shanghai University of Medicine and Health Sciences, Shanghai, China; 2 Shanghai Novamab Biopharmaceuticals Co., Ltd., Shanghai, China; 3 Shanghai Donghai Geriatric Nursing Hospital, Shanghai, China

**Keywords:** IL-5, Eosinophilic asthma, Trivalent nanobody, Long-acting

## Abstract

**Background:**

Eosinophilic asthma is a common subtype of severe asthma with high morbidity and mortality. The cytokine IL-5 has been shown to be a key driver of the development and progression of disease. Although approved monoclonal antibodies (mAbs) targeting IL-5/IL-5R have shown good safety and efficacy, some patients have inadequate responses and frequent dosing results in medication nonadherence.

**Results:**

We constructed a novel trivalent bispecific nanobody (Nb) consisting of 3 VHHs that bind to 2 different epitopes of IL-5 and 1 epitope of albumin derived from immunized phage display libraries. This trivalent IL-5-HSA Nb exhibited similar IL-5/IL-5R blocking activities to mepolizumab (Nucala), an approved targeting IL-5 mAb. Surprisingly, this trivalent Nb was 58 times more active than mepolizumab in inhibiting TF-1-cell proliferation. In primate studies, the trivalent IL-5-HSA Nb showed excellent pharmacokinetic properties, and peripheral blood eosinophil levels remained significantly suppressed for two months after a single dose. In addition, the trivalent IL-5-HSA Nb could be produced on a large scale in a *P. pastoris X-33* yeast system with high purity and good thermal stability.

**Conclusions:**

These findings suggest that the trivalent bispecific IL-5-HSA Nb has the potential to be a next-generation therapeutic agent targeting IL-5 for the treatment of severe eosinophilic asthma.

**Graphical Abstract:**

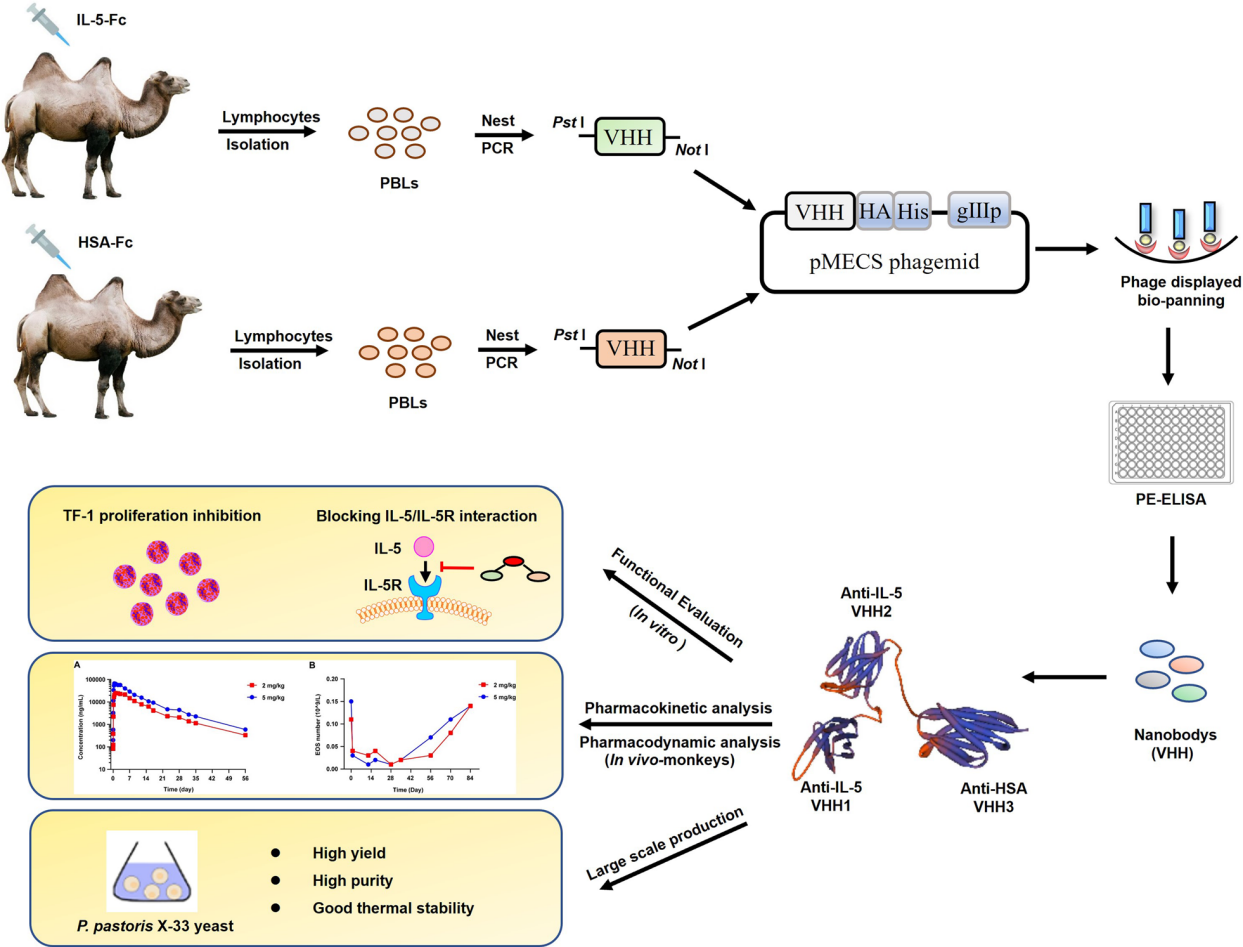

**Supplementary Information:**

The online version contains supplementary material available at 10.1186/s12931-022-02240-1.

## Background

Eosinophilic asthma is a common subtype of severe asthma with high morbidity and mortality [1]. It is characterized by the persistence of eosinophils in the lung and sputum, resulting in inflammation and swelling in the airways [2]. Eosinophil abnormalities have been shown to be associated with the production and release of some soluble inflammatory factors, of which IL-5 is the key factor for the growth and differentiation of eosinophils in bone marrow and infiltration and activation in tissues [3–5]. IL-5 expression has been shown to be increased in bronchial biopsies in patients with eosinophilic asthma [6, 7]. Additionally, the level of IL-5 in the blood and lung correlates with disease severity [8–10]. Therefore, blocking the IL-5/IL-5R pathway is considered to be an attractive strategy for the treatment of eosinophilic asthma.

Several mAbs targeting the IL-5/IL-5R pathway have been approved by the FDA for the treatment of severe eosinophilic asthma [11]. Mepolizumab (Nucala) is the first approved monoclonal antibody (mAb) targeting IL-5, blocking the binding of IL-5 to IL-5Rα, which reduces blood eosinophil counts and exacerbations of asthma and improves asthma control [12]. Reslizumab, another approved IL-5 humanized mAb, showed similar therapeutic efficacy to mepolizumab in severe eosinophilic asthma [13]. However, in a study of 10 patients with oral corticosteroid-dependent asthma, reslizumab was more effective than mepolizumab in reducing sputum eosinophilia [14]. Benralizumab, the only approved mAb targeting IL-5R, also reduces exacerbation frequency and improves the quality of life of severe asthma patients [15]. Unlike antibodies that target IL-5, benralizumab reduces eosinophils more efficiently via antibody-dependent cell-mediated cytotoxicity, resulting in the improvement of lung function or asthma control [16]. In addition, GSK3511294, another IL-5 mAb, with the same antigen-binding epitope as mepolizumab, but with higher affinity and longer half-life, is currently in phase 3 clinical investigation for the treatment of severe eosinophilic asthma [17]. These findings suggest that biologics targeting IL-5/IL-5R with less frequent dosing, higher tissue penetration, and novel mechanisms of action remain needed for the treatment of severe eosinophilic asthma.

Nanobodies (Nbs) are single-domain antibody fragments (VHHs) derived from natural heavy chain-only antibodies in the Camelidae family of mammals [18]. Unlike traditional antibodies composed of 2 heavy chains and 2 light chains, the molecular weight of Nbs is as small as 15 kD, which enables Nbs to exhibit excellent stability and tissue penetration [19]. Importantly, this unique structure and stability make Nbs the ideal building blocks for the development of bispecific or multispecific antibodies [20]. Several reported Nb-based bispecific or trispecific Nbs are in clinical development for the treatment of tumours and autoimmune diseases [21–24].

Here we constructed a novel heterobivalent Nb consisting of 2 Nbs targeting different epitopes of IL-5. To prolong the half-life in vivo, this heterobivalent Nb was loaded with a VHH targeting albumin, resulting in a new trivalent Nb. This trivalent Nb showed significantly better activity than mepolizumab, a traditional mAb targeting IL-5, in inhibiting TF-1-cell proliferation. In primate studies, this trivalent Nb showed excellent pharmacokinetic properties, and peripheral blood eosinophil levels remained significantly suppressed for 2 months after a single dose. Moreover, the large production of the trivalent Nb using the *P. pastoris* expression system has a significant cost advantage over mAb. These findings suggest that this trivalent Nb has the potential to be a next-generation therapeutic agent targeting IL-5 for the treatment of severe eosinophilic asthma, with better efficacy and less frequent dosing than current anti-IL-5/IL-5R therapies.

## Materials and methods

### Cell lines

HEK 293T, HEK 293F and TF-1 cells were obtained from the American Type Culture Collection (ATCC). HEK 293T and HEK 293F cells were grown in Dulbecco’s modified Eagle’s medium (DMEM) (Gibco, Grand Island, NY, USA) and FreeStyle™ 293 medium (Invitrogen, Carlsbad, CA, USA) respectively, supplemented with 10% FBS (Gibco) and 1% penicillin–streptomycin (10,000 U/mL) (Gibco). TF-1 cells were cultured in RPMI 1640 containing 20% FBS and 50 U/mL GM-CSF (Thermo Fisher Scientific).

### Antigen immunization and library construction

The IL-5 gene was amplified by PCR and inserted into the pFUSE vector (Invitrogen). IL-5-Fc was expressed in HEK-293F cells and purified with protein A affinity chromatography. Two young male Bactrian camels received seven immunizations with IL-5-Fc mixed with Freund’s adjuvant (Sigma-Aldrich, St Louis, MO, USA) at weekly intervals. Thereafter, peripheral blood lymphocytes (PBLs) were collected from 100 mL camel blood and a phage display library containing genes coding for the VHH was then produced according to established protocols [25, 26]. The library capacity and correct insert rates were calculated. All procedures were conducted according to the National Institutes of Health Guide for the Care and Use of Laboratory Animals.

### Nbs screening, expression and purification

Human IL-5-specific Nbs were screened through phage display biopanning and periplasmic extract ELISA, according to previously described protocols [21, 23]. After sequencing the selected positive clones, the VHH fragments with different complementary determining regions 3 (CDR3) cloned into the pMECS vector were transformed into the nonsuppressor *Escherichia coli* strain WK6 cells and purified using affinity chromatography on Ni–NTA affinity columns (Qiagen, Hilden, Germany).

### Preparation of bivalent IL-5 Nb, trivalent IL-5-HSA Nb and Nucala analogue

Bivalent IL-5 Nb consists of two IL-5 Nbs with different antigen recognizing epitope sites, while trivalent Nb consists of two IL-5 Nbs and HSA Nb. The recombinant antibody sequences were amplified and inserted into the pcDNA3.1+ vector. Thereafter, the recombinant vectors were transfected into HEK-293F cells, and the bivalent IL-5 Nb and the trivalent IL-5-HSA Nb were purified by Ni–NTA affinity chromatography. For preparation of the positive control antibody Nucala analogue, the DNA sequences of its heavy chain and light chain were synthesized and cloned into pcDNA3.1+ vector, and its expression and purification were similar to those of bivalent and trivalent Nb.

### Determination of the affinity of IL-5 Nb for IL-5

The kinetics of IL-5 Nb binding to recombinant hIL-5 antigen were evaluated by biolayer interferometry (BLI) on an Octet Red96e (ForteBio, Menlo Park, CA, USA). Briefly, the diluted IL-5 Nbs (5 μg/mL) were coupled to protein A biosensors and then incubated with a series of diluted hIL-5 antigens, followed by dissociation in PBST buffer. The binding curve fitting was performed in 1:1 homogeneous fitting mode by ForteBio Data Analysis 9.0 software. The association and dissociation rates were monitored and the equilibrium dissociation constant (KD) was calculated.

### Recognition specificity analysis

Human IL-5-Fc, cynomolgus IL-5-Fc and mouse IL-5-Fc were expressed and purified in HEK 293F cells. 5 μg/mL proteins (human IL-5-Fc, cynomolgus IL-5-Fc, mouse IL-5-Fc and IgG1) were coated onto microtiter plates at 4 °C overnight. After blocking with 1% bovine serum albumin (BSA) for 2 h, 10 μg/mL IL-5 Nb was added and incubated at room temperature for 1 h. Next, the plates were incubated with mouse anti-HA antibody (Biolegend, San Diego, CA, USA), followed by anti-mouse IgG-alkaline phosphatase (Sigma-Aldrich). The absorbance at 405 nm was read on a microplate reader (Bio-Rad, Hercules, CA, USA).

### IL-5/IL-5R blocking activity assay

The HEK 293T-IL-5R stable cell line was constructed using a lentiviral packaging system. To determine the activity of IL-5 Nbs blocking IL-5/IL-5R, HEK 293T-IL-5R stable cells were incubated with purified IL-5-Fc labelled with biotin and a gradient concentration of IL-5 Nbs or Nucala analogue, followed by staining with streptavidin-PE (eBioscience, San Diego, CA, USA). Then, the signals were measured by FACS (BD Biosciences, Franklin Lakes, New Jersey, USA), and the data were analysed with FlowJo V10 software. The 50% inhibitory concentration (IC_50_) was calculated.

### Cell proliferation assay

The proliferation of TF-1 cells was determined with a CCK-8 assay (Dojindo, Kumamoto, Japan). The TF-1 cells were seeded into 96-well plates at approximately 1 × 10^5^ cells per well overnight. Then, the TF-1 cells were treated with a series of diluted concentrations of IL-5 Nb and incubated at 37 °C for 72 h. Thereafter, 20 μL CCK-8 solution was added and incubated for an additional 4 h at 37 °C. The reaction signals were detected at 450 nm with a microplate reader. The IC_50_ was calculated.

### Pharmacokinetic study in non-human primates

Female cynomolgus monkeys were housed individually in stainless-steel cages with suitable specifications, and reared in humidity-controlled (40% to 70%) and temperature-controlled (22 ± 4 °C) environments on a 12 h light/dark cycle with free access to water and food. One monkey enrolled in the high-dose group was given treatment of 5 mg/kg trivalent IL-5-HSA Nb, and another monkey enrolled in the low-dose group was given treatment of 2 mg/kg trivalent IL-5-HSA Nb. Subcutaneous infusion was administered for approximately 10 min with a given rate of 0.5 mL/kg/min. Blood samples were collected every 7 days and continued for three months. Then, the plasma concentrations of trivalent IL-5-HSA Nb were detected through SEC-HPLC analysis. The experiments were approved by the Animal Experimental Ethics Committee of Shanghai University of Medicine and Health Sciences.

### Measurement of eosinophils (EOS)

EOS were purified from 30 mL monkey peripheral blood samples though an immunomagnetic negative-selection approach with the MACSxpress Eosinophil Isolation Kit (Miltenyi Biotec, Bergisch Gladbach, Germany), and the residual erythrocytes were removed with the Erythrocyte Depletion Kit (Miltenyi Biotec). Then, the number of harvested EOSs suspended in PBS was quantified on a Countess II FL automatic cell counter (Thermo Fisher Scientific).

### Large-scale production of trivalent IL-5-HSA Nb

The fused fragment encoding trivalent IL-5-HSA Nb was ligated into the pPICZɑA vector, and then the recombinant vector was transformed into *P. pastoris* X-33 competent cells through electroporation. After identifying the positive clone with high expression efficiency, large-scale expression was carried out in a 100-L fermenter. During the fermentation process, the dissolved oxygen (DO) was maintained at 25% by supplementation with glycerol and methanol, and the wet cell weights and the protein titers were detected every 20 h within 160 h. Thereafter, the fused protein was further purified through a Ni–NTA affinity column, followed by Capto Butyl column and Superdex 200 Increase GL columns on AKTA pure 150 (GE Healthcare, Madison, WI, USA). The nonreductive form expression of trivalent IL-5-HSA Nb was identified by SDS-PAGE analysis, and its purity was detected by size exclusion chromatography-high-performance liquid chromatography (SEC-HPLC) using a Waters Acquity Arc system with AdvanceBio SEC 130A Columns (Agilent Technologies, Palo Alto, CA, USA).

### Thermostability analysis

SEC-HPLC and cation-exchange chromatography (CEX-HPLC) were employed to analyse the thermostability of trivalent IL-5-HSA Nb. Briefly, the trivalent IL-5-HSA Nb was diluted to 1 mg/mL and incubated at 2–8 °C or 25 °C for 1 month or even under 3 freeze-and-thaw cycles. After incubation, the stability of Nb was measured by SEC-HPLC and CEX-HPLC analyses. CEX-HPLC was performed on a Dionex Ultimate 3000 RSLC system (Thermo Fisher Scientific) with a BioMab NP5 PK column (Agilent Technologies).

### Statistical analysis

Statistical analyses were performed using GraphPad Prism 6 software. Data were expressed as the mean ± SD. The statistically significant differences between groups were analysed by two-way ANOVA with Holm‒Sidak multiple comparisons test. *P* < 0.05 was considered to be significant.

## Results

### Identification of Nbs specific binding to IL-5

**Fig. 1:**
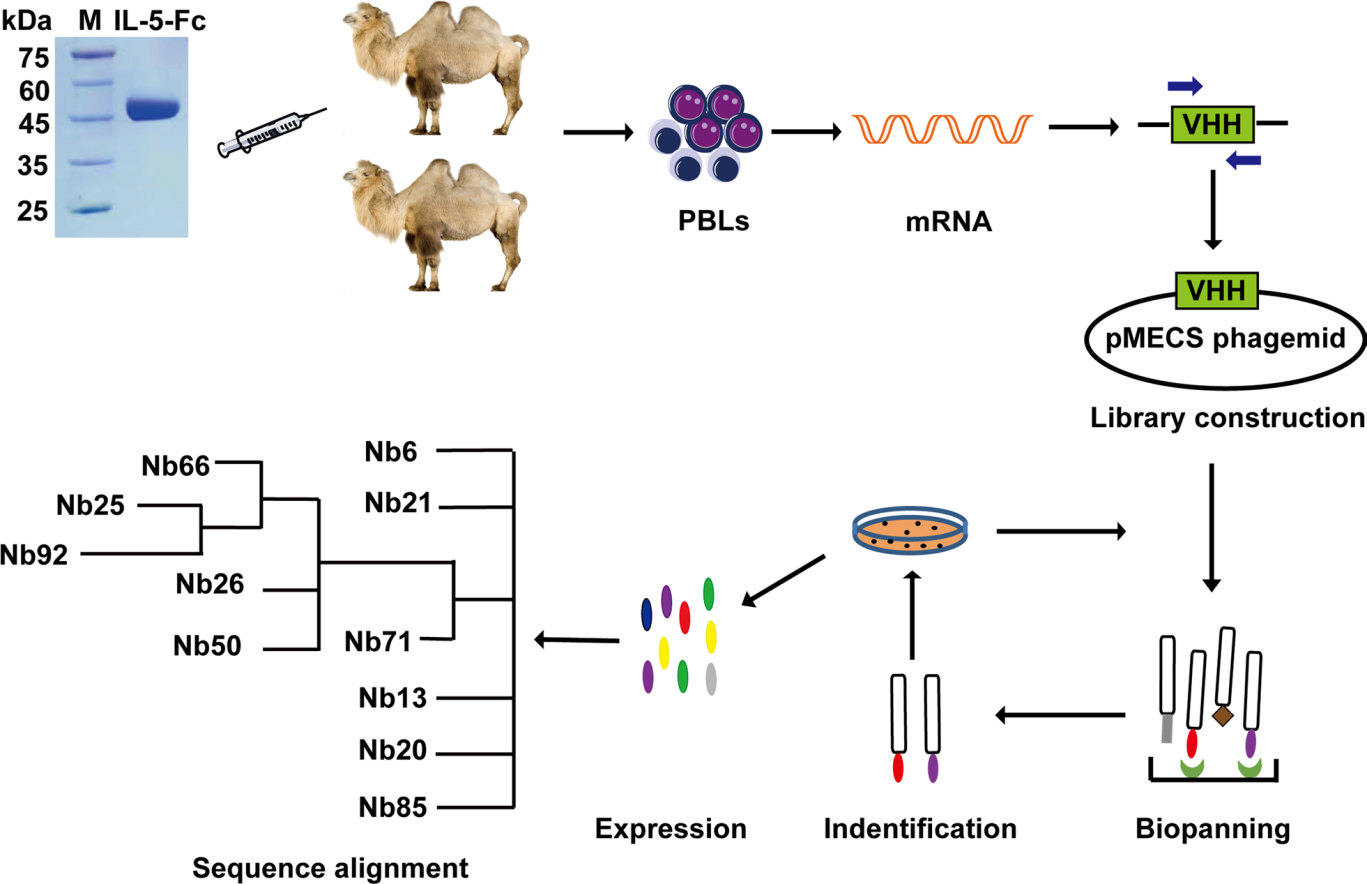
Schematic depicting the immunization and screening strategy used to isolate IL-5 Nbs. PBLs, peripheral blood lymphocytes. The cropping gel of purified IL-5-Fc antigen is displayed

To obtain Nbs with high specificity, affinity and diversity, two camels were immunized with IL-5 fused to Fc (IL-5-Fc) (Fig. 1). After seven injections at an interval of five days, the peripheral blood of each immunized camel was collected to construct the phage-displayed library. The two library capacities were 2.1 × 10^8^ colony-forming units (CFU) and 6.4 × 10^8^ CFU, and the insertion rates of two libraries were 95.8% and 91.7% respectively, indicating high quality and good diversity of both libraries (Additional file 1: Fig. S1). After three rounds of phage display biopanning, the fold enrichment of IL-5-specific VHHs derived from the two libraries reached 66 times and 120 times, respectively (Additional file 2: Fig. S2). Through periplasmic extract ELISA and sequencing analysis, 11 distinct Nbs with substantial sequence diversity were screened. The entire schedule is illustrated in Fig. 1, and the original, full-length gel images of IL-5-Fc antigen, amplified VHH and library insert rate are displayed in Additional file 3: Fig. S3A–E.

### Generation of heterobivalent IL-5 Nb with high affinity, specificity and blocking activity

**Fig. 2:**
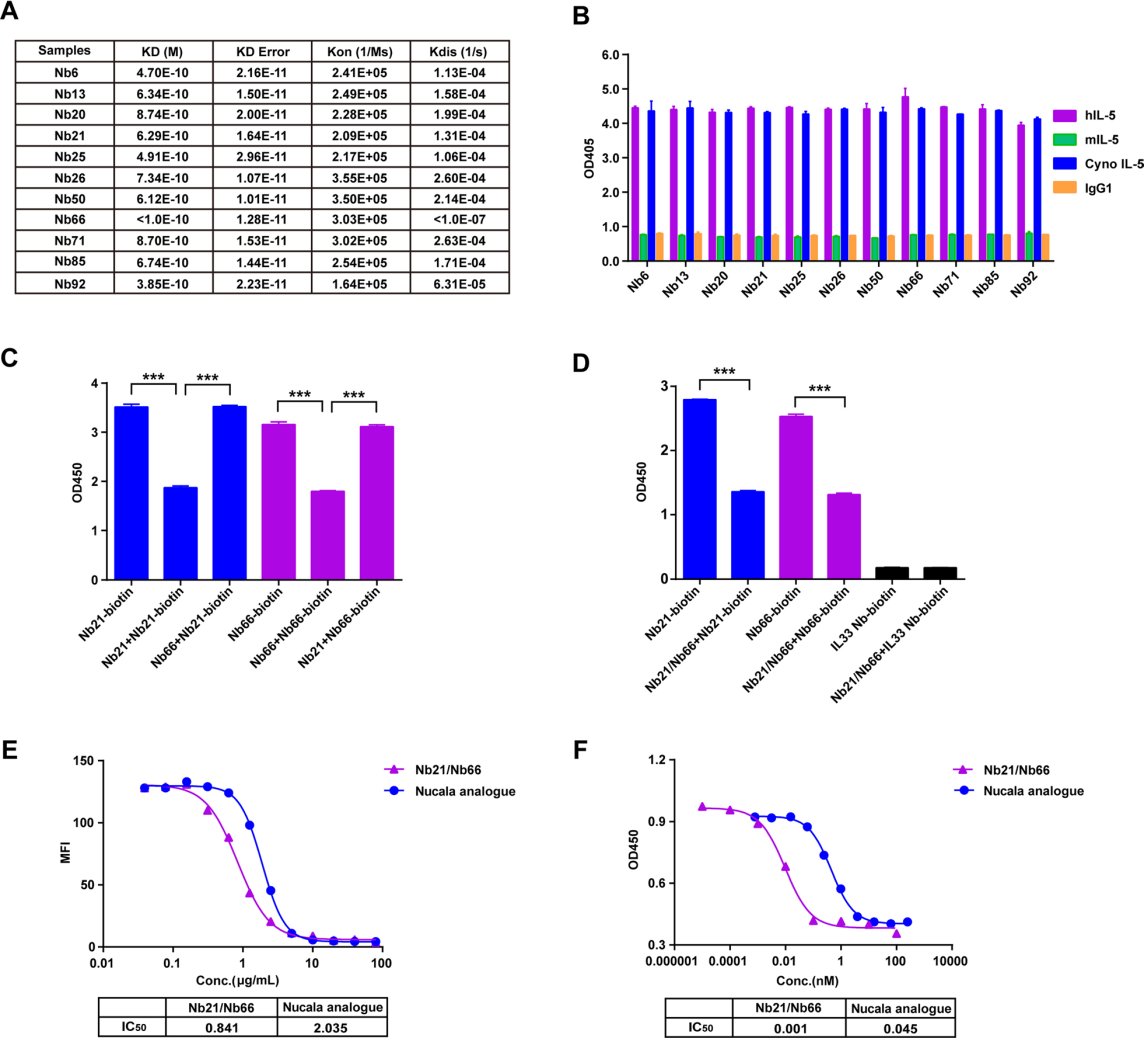
The generation of heterobivalent Nb21/Nb66. **A** The affinity of 11 IL-5 Nbs was detected by biofilm interferometry (BLI). **B** The recognition specificity of 11 IL-5 Nbs to different species antigens was determined by ELISA, with IgG1 as a negative control. **C** The competition binding activity of Nb66 or Nb21 towards the binding between Nb21-biotin and IL-5 or Nb66-biotin and IL-5 was detected by ELISA. **D** The recognition specificity of Nb21/Nb66 towards Nb21-biotin-IL-5 or Nb66-biotin-IL-5 binding sites, with IL33-Nb biotin as a negative control. The experiments were performed in triplicate. The data were presented as mean ± SD. ***P < 0.001. **E** The blocking effect of the Nb21/Nb66 or Nucala analogue on the interaction between IL-5 and IL-5R was determined by flow cytometry. MFI: median fluorescence intensity. **F** The inhibitory effect of the Nb21/Nb66 or Nucala analogue on TF-1-cells proliferation was determined by the CCK8 assay. The IC_50_ was calculated

After screening 11 IL-5-specific Nbs through phage display biopanning and periplasmic extract ELISA, we further determined their characteristics, including affinity and specificity. As indicated in Fig. 2A, all of these IL-5 Nbs candidates purified through microbial expression exhibited excellent affinities towards IL-5. Additionally, they showed high recognition species specificity towards human IL-5 and cynomolgus monkey IL-5, instead of mouse IL-5 (Fig. 2B). To generate IL-5 bivalent Nb with enhanced functional activity, humanized IL-5 Nbs with different antigen recognition epitopes were screened through the competition binding assay. As shown in Fig. 2C, Nb66 did not influence the binding between Nb21-biotin and IL-5. In parallel with this, Nb21 also exhibited no competing inhibitory effect towards Nb66-biotin binding with IL-5, indicating that Nb21 and Nb66 recognize different epitope sites. Furthermore, heterobivalent Nb21/Nb66 was constructed, and the results showed that the bivalent IL-5 Nb was competitively bound to the Nb21-IL-5 or Nb66-IL-5 interacting sites, implying that the bivalent IL-5 Nb specifically recognizes two different epitope sites (Fig. 2D).

To evaluate the blocking effect of Nb21/Nb66, a neutralization assay was performed. Serial dilutions of Nb21/Nb66 and Nucala were incubated with IL-5R stable-expressing cells in the presence of IL-5-Fc protein. The results indicated that Nb21/Nb66 had higher blockage activity towards the IL-5/IL-5R interaction, than the Nucala analogue. (IC_50_: 0.841 μg/mL vs. 2.305 μg/mL) (Fig. 2E). Additionally, Nb21/Nb66 exhibited better inhibitory activity towards the proliferation of IL-5-dependent TF-1 cells than the Nucala analogue (IC_50_: 0.001 μg/mL vs. 0.045 μg/mL) (Fig. 2F). These findings suggest that bivalent Nb21/Nb66 exhibits high IL-5/IL-5R blockage activity and TF-1 inhibitory activity.

### Long-acting trivalent bispecific Nb targeting both IL-5 and HSA was prepared

**Fig. 3:**
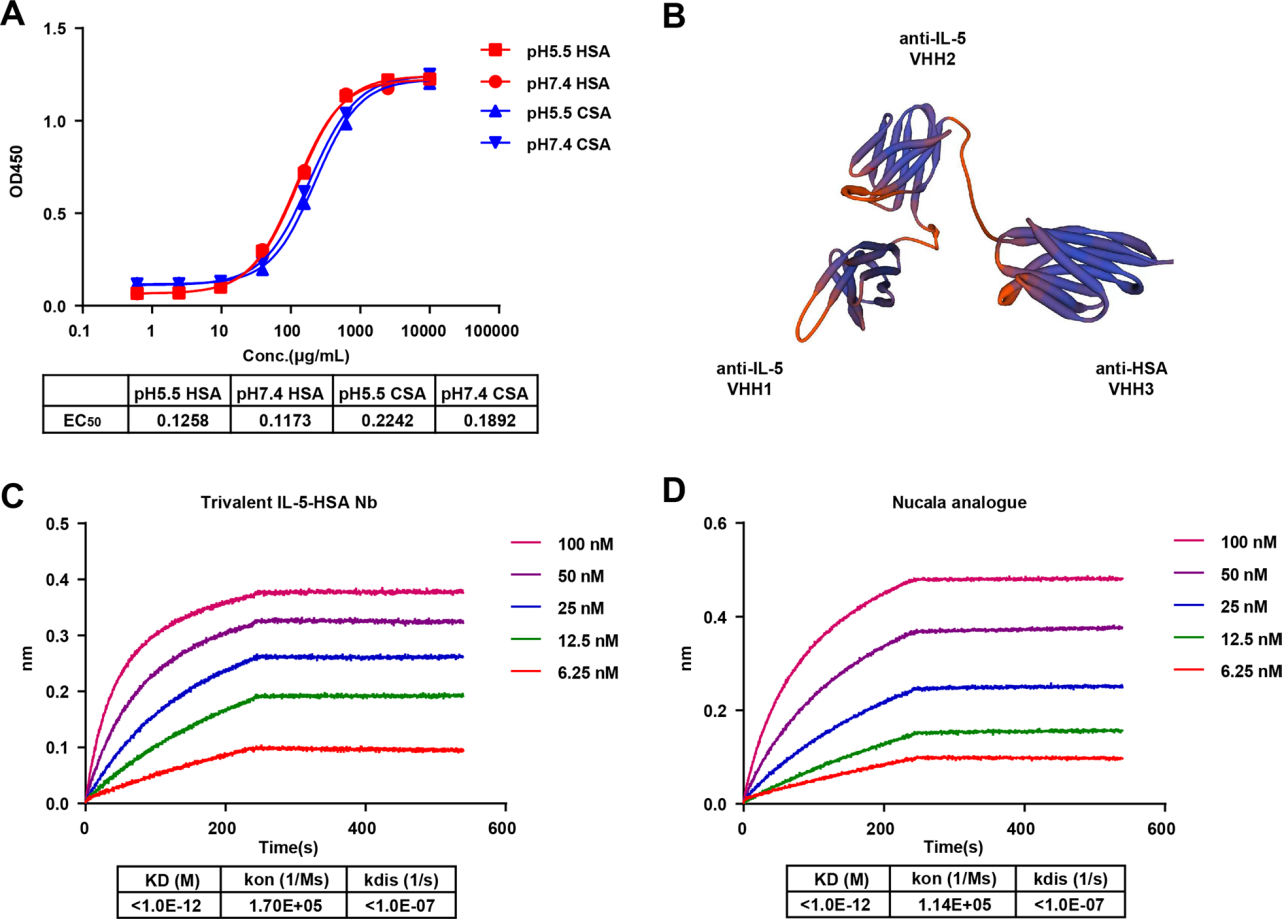
Construction of the trivalent bispecific IL-5-HSA Nb. **A** The binding activity of HSA Nb towards human albumin and cynomolgus monkey albumin (CSA) at pH 5.5 and pH 7.4 was determined by ELISA. **B** The structure of trivalent bispecific IL-5-HSA Nb. **C-D** The affinity of trivalent IL-5-HSA Nb and Nucala analogue to IL-5 was detected by Fortebio

Considering that serum albumin fusion is a potential means to prolong the half-life of drugs, but albumin is difficult to obtain and is easily contaminated when extracted from blood, we prepared anti-HSA Nb with high specificity and affinity from camel-immunized libraries. Thereafter, two pH gradients of 5.5 and 7.4 were set, under which the neonatal Fc receptor (FcRn) prolongs the life cycle of HSA. As shown in Fig. 3A, the HSA Nb exhibited good binding activity towards human serum albumin (HSA) and monkey serum albumin (CSA) at pH 5.5 and pH 7.4, with EC_50_ values towards HSA of 0.1258 μg/mL and 0.1173 μg/mL, and EC_50_ values towards CSA of 0.2242 μg/mL and 0.1892 μg/mL. Therefore, the screened HSA Nb displayed high binding activity within the pH range of FcRn works (Fig. 3A).

To facilitate the long-lasting effect of anti-IL-5 Nb21/Nb66, we established anti-IL-5/HSA trivalent constructs. The structure of trivalent bispecific Nbs, which consist of IL-5 Nb66 (VHH1), IL-5 Nb21 (VHH2) and HSA Nb (VHH3), is illustrated in Fig. 3B. In the affinity assay, we showed that the KD value of trivalent IL-5-HSA Nb was lower than 1.0E − 12 M, which implies that trivalent IL-5-HSA Nb exhibits high affinity towards IL-5 to the same extent as Nucala analogue (Fig. 3C and D).

### The trivalent IL-5-HSA Nb shows high blockage activity and TF-1 inhibitory activity

**Fig. 4:**
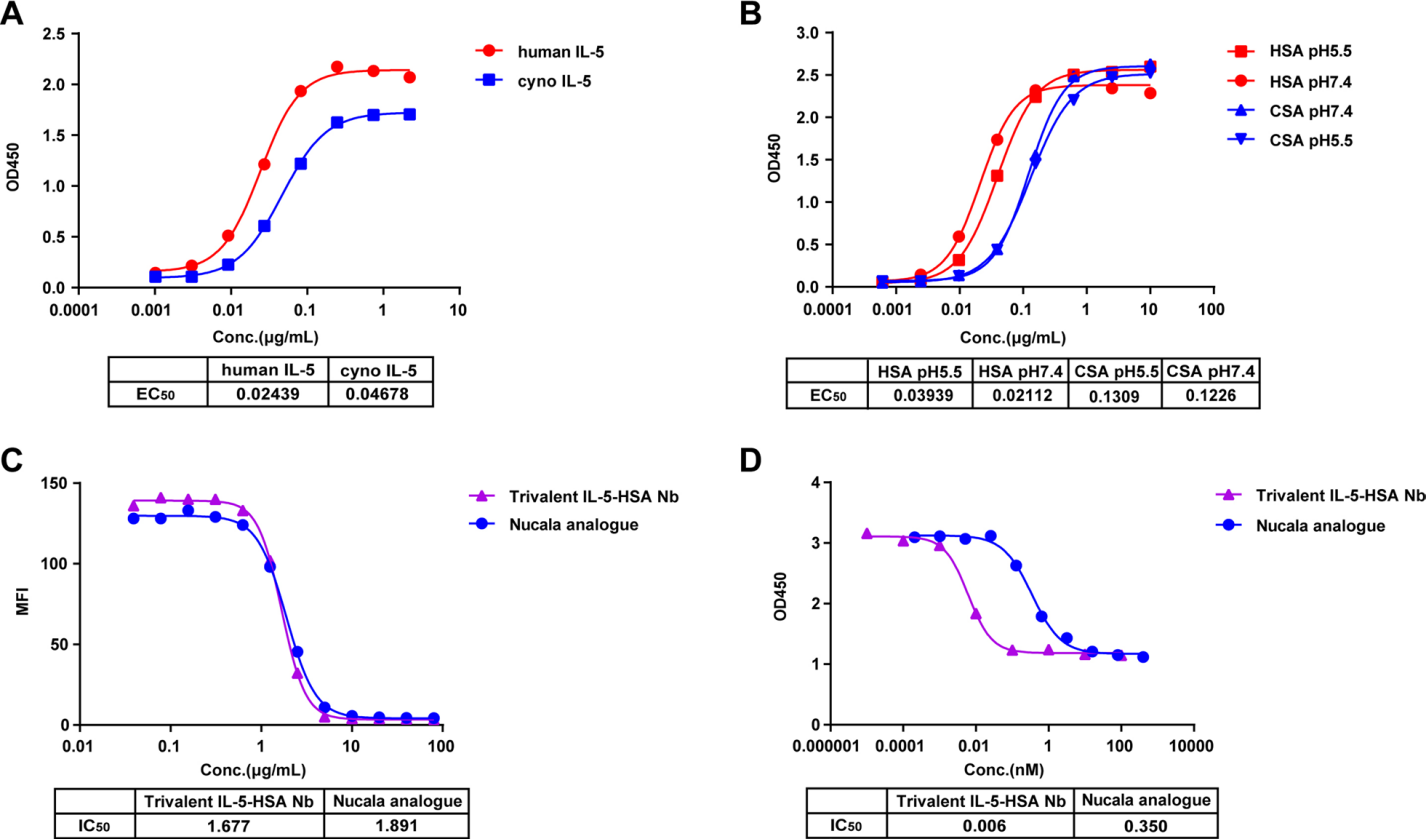
The functional activity determination of the trivalent IL-5-HSA Nb. **A** The binding activity of the trivalent IL-5-HSA Nb towards human IL-5 and cyno IL-5. **B** The binding activity of the trivalent IL-5-HSA Nb towards HSA and CSA at pH 5.5 and pH 7.4. The binding activity was detected by ELISA. **C** The blocking effect of trivalent IL-5-HSA Nb or Nucala analogue on the IL-5/IL-5R interaction was determined by flow cytometry. **D** The inhibitory effect of trivalent IL-5-HSA Nb or Nucala analogue on TF-1-cell proliferation was determined by the CCK8 assay

To assess the function of trivalent IL-5-HSA Nb, the binding ability towards IL-5 and HSA in human and monkey species was determined. The results showed that trivalent IL-5-HSA Nb exhibited high binding activity to both human IL-5 and cyno IL-5 with EC_50_ values of 0.024 μg/mL and 0.047 μg/mL respectively (Fig. 4A). Similarly, trivalent IL-5-HSA Nb displayed good binding activity towards HSA and CSA at pH 5.5 and pH 7.4, with EC_50_ values towards HSA of 0.039 μg/mL and 0.021 μg/mL, respectively, and EC_50_ values towards CSA of 0.131 μg/mL and 0.123 μg/mL, respectively (Fig. 4B). These results demonstrated that trivalent IL-5-HSA Nb could target both IL-5 and HSA.

We next evaluated the blockage activity of trivalent IL-5-HSA Nb towards the IL-5/IL-5R interaction and its inhibitory activity towards TF-1 cells. As shown in Fig. 4C, the trivalent IL-5-HSA Nb exhibited similar IL-5/IL-5R blocking activities to the Nucala analogue (IC_50_: 1.677 μg/mL vs. 1.891 μg/mL). In addition, in the biological functional analysis, the trivalent IL-5-HSA Nb treated group demonstrated a significant inhibitory effect on TF-1-cell proliferation, compared to the Nucala analogue-treated group (IC_50_: 0.006 nM vs. 0.350 nM), of note, the IC_50_ fold difference between the two groups reached 58 (Fig. 4D). Taken together, these results suggest that the trivalent Nb cotargeting IL-5 and HSA showed higher functional activity than the Nucala analogue.

### The trivalent IL-5-HSA Nb displays a long half-life and eosinophil inhibition in vivo

**Fig. 5:**
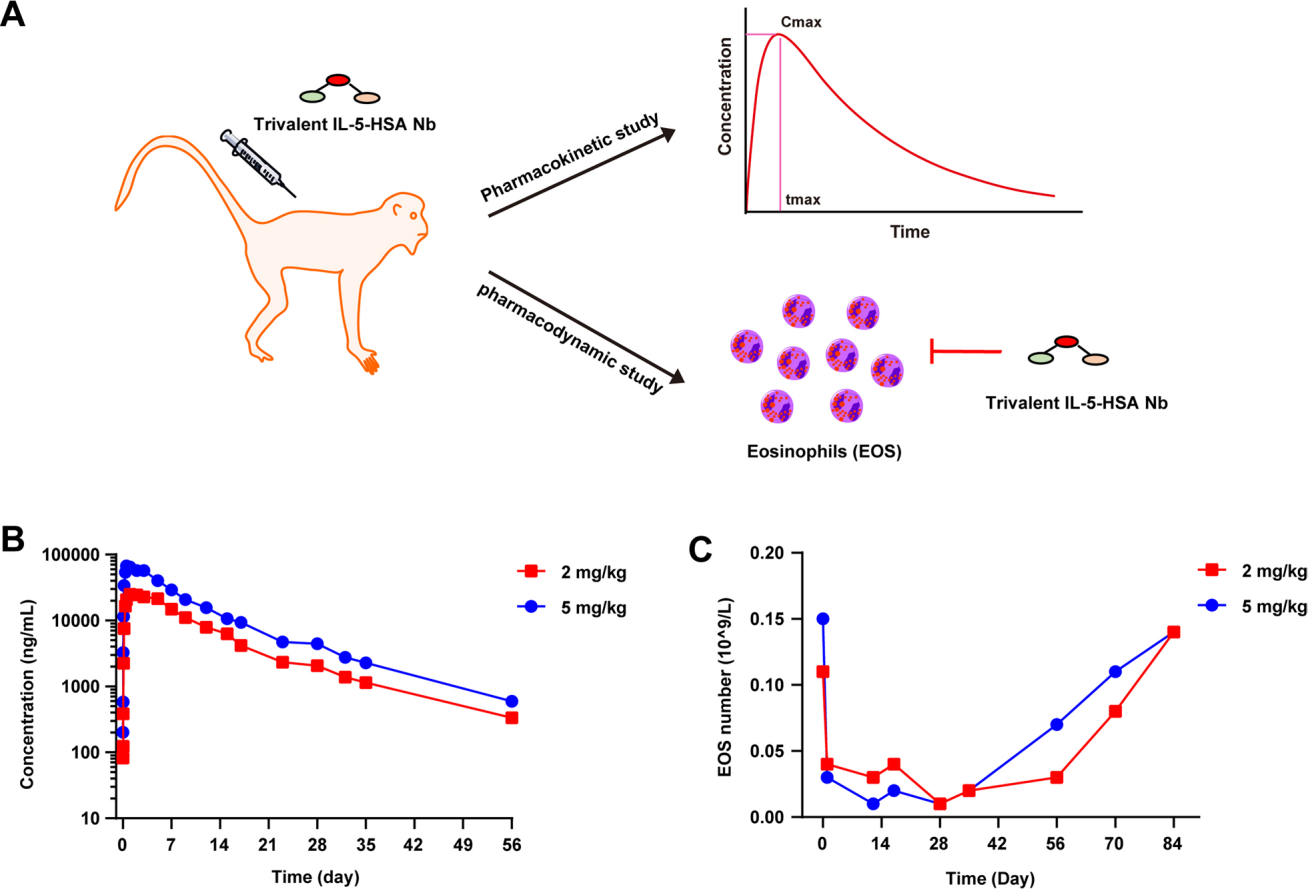
The pharmacokinetic and pharmacodynamic effects of the trivalent IL-5-HSA Nb in cynomolgus monkeys. **A** Schematic representation of its pharmacokinetic and pharmacodynamic study. **B** The pharmacokinetic effect of the trivalent IL-5-HSA Nb in cynomolgus monkeys with the primary doses of 5 mg/kg and 2 mg/kg. The plasma concentration of trivalent IL-5-HSA Nb was measured within 56 days. **C** The inhibitory effect of the trivalent IL-5-HSA Nb on eosinophil (EOS) numbers was determined within 84 days

To confirm that the trivalent IL-5-HSA Nb could play a long-lasting role and inhibit eosinophils in vivo, we studied its pharmacokinetics and pharmacodynamics in two cynomolgus monkeys for approximately two months and three months, respectively. The schematic representation is illustrated in Fig. 5A. As shown in Fig. 5B, the serum pharmacological concentration decrease of the trivalent IL-5-HSA Nb with priming doses of 5 mg/kg and 2 mg/kg showed a consistent tendency, and the half-life period of the trivalent IL-5-HSA Nb reached approximately 12 days. Furthermore, its serum concentration showed slow clearance and could be retained for over 56 days in vivo.

Considering that IL-5 is specific for eosinophils, and plays an essential role in inducing eosinophil growth, differentiation, and survival, we next explored the inhibitory role of the trivalent IL-5-HSA Nb on eosinophils in cynomolgus monkeys. As expected, treatment of cynomolgus monkeys with trivalent IL-5-HSA Nb reduced eosinophil numbers at doses of 5 mg/kg and 2 mg/kg. As indicated in Fig. 5C, the eosinophil numbers were reduced to 1 × 10^7^/L at Day 28, and the inhibitory rate reached 93.3%. Thereafter, the eosinophil numbers were gradually restored to normal levels after approximately 84 days. Overall, combined with the pharmacokinetic and pharmacodynamic effects of the trivalent IL-5-HSA Nb, its therapeutic effect could be expected to last for 2–3 months. Thus, the clinical administration duration of the trivalent IL-5-HSA Nb could be significantly extended.

### The trivalent IL-5-HSA Nb exhibits good developability

**Fig. 6:**
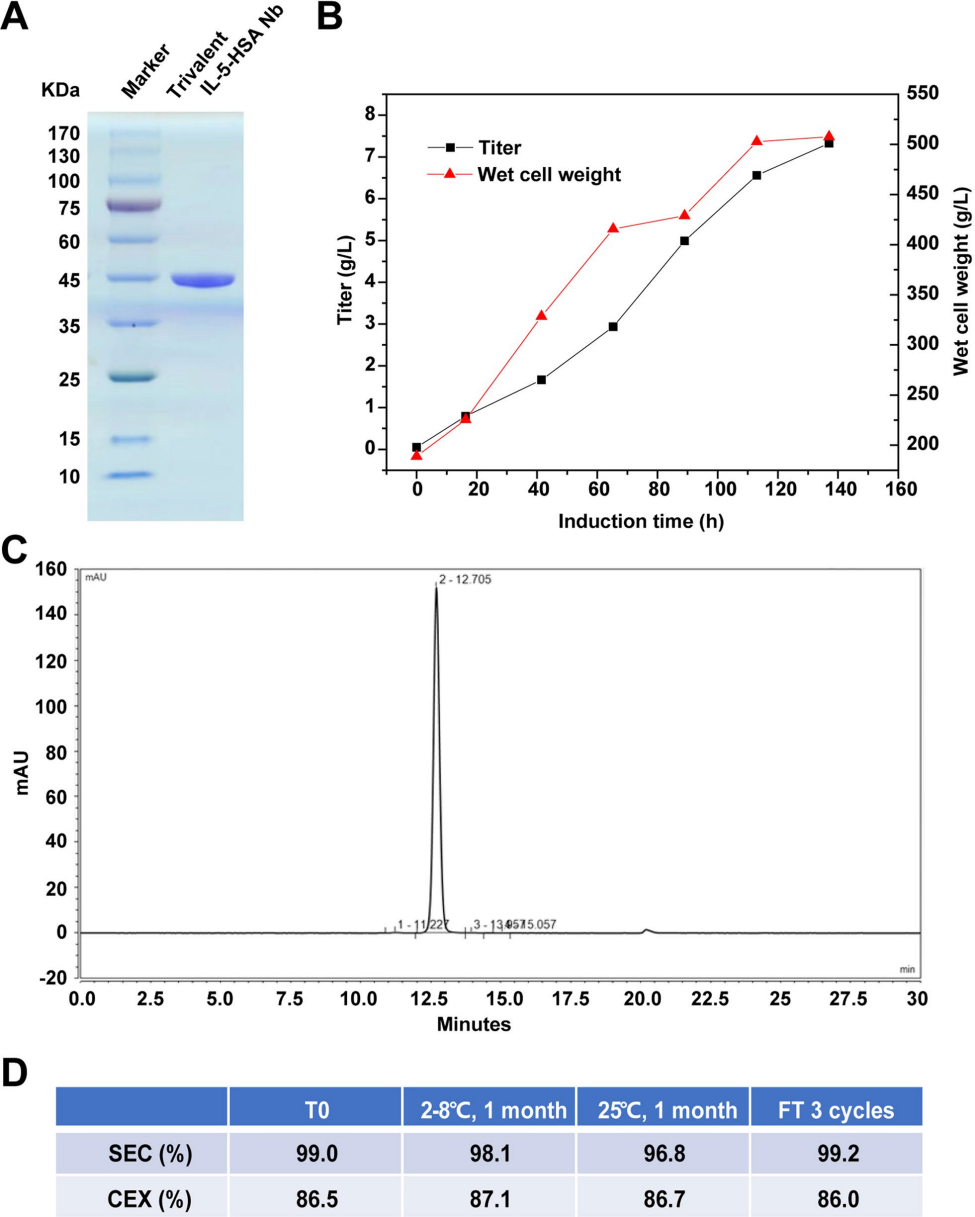
Large-scale production of the trivalent IL-5-HSA Nb and its preliminary developability analysis. **A** The protein expression of the trivalent IL-5-HSA Nb was detected through SDS-PAGE, followed by affinity chromatography, hydrophobic chromatography and molecular sieve chromatography. The cropping gel of purified trivalent IL-5-HSA Nb is displayed. **B** The protein titer and wet cell weight in the fermentation tank were both measured at the indicated times. **C** The purity of the trivalent IL-5-HSA Nb was determined through SEC-HPLC analysis. **D** The stability of the trivalent IL-5-HSA Nb under different temperatures and 3 freeze‒thaw cycles was detected by SEC-HPLC and CEX-HPLC

Based on the above functional studies of the trivalent IL-5-HSA Nb in vivo and in vitro, we further evaluated its developability. The expression of the trivalent IL-5-HSA Nb was induced in a yeast system and it was purified through affinity chromatography, hydrophobic chromatography and molecular sieve chromatography. Its reductive form is displayed in Fig. 6A, and its original, full-length gel image is displayed in Additional file 3: Fig. S3F. As indicated in Fig. 6B and C, the wet cell weight reached to 500 g/L, and the protein yield of the trivalent IL-5-HSA Nb reached 7.5 g/L after 140 h in a 100 L fermenter (Fig. 6B). Meanwhile, the purity was 99.5% according to HPLC analysis (Fig. 6C). After extensive formula screening and optimization, it was finally confirmed that a preparation could make the antibody have high solubility (100 mg/mL) and good stability (Fig. 6D). Thermostability analysis showed that the trivalent IL-5-HSA Nb remained stable after incubation at 2–8 °C or 25 °C for 1 month or even under 3 freeze–thaw cycles. Of note, the proportion of the monomeric form of the trivalent IL-5-HSA Nb remained over 96%. In summary, these results imply that we obtained trivalent IL-5-HSA Nb with high yield, high purity and good thermal stability in a yeast expression system.

## Discussion

To overcome the limitations of frequent dosing and the presence of non-responsive patients to the approved mAbs targeting IL-5, we aimed to design a novel molecule with high avidity that could bind to different epitopes of IL-5. The construction of biparatopic antibody is a suitable strategy to achieve this goal since the binding caused by two points of contact between the biparatopic antibody and the antigen is much less likely to be reversible than single point of contact of the mAb with the antigen. In order to construct a novel biparatopic antibody, we screened 11 Nbs targeting IL-5 from a camel-derived phage nanolibrary with KD values ranging from < 1 × 10^–10^ to 8.7 × 10^–10^, which are similar to approved mAbs [27]. Furthermore, we obtained two Nbs Nb21 and Nb66 targeting different epitopes of human IL-5. The heterobivalent Nb21/Nb66 showed the better inhibitory effect on TF-1-cells proliferation compared to their homobivalent counterparts (Additional file 4: Fig. S4). Similarly, several reported bispecific antibodies targeting different epitopes of the same antigen have also shown significant functional advantages over native antibodies [28–30]. Of note, Nb21/Nb66 showed slightly superior blocking activity compared to mepolizumab (Nucala), with 0.8 μg/mL versus 2.0 μg/mL. However, Nb21/Nb66 had a 45-fold higher inhibitory activity than mepolizumab against the proliferation of TF-1 cells, representing the ability to inhibit eosinophils in vivo. Therefore, our findings might suggest that the better performance of the biparatopic antibody Nb21/Nb66 in the inhibition of cell function is not completely due to the difference in avidity, compared to the approved mepolizumab. Moreover, different antigen-binding epitopes or structural differences in the antigen–antibody complexes may be the key contributors for the differences in inhibition of eosinophil proliferation. However, the precise molecular mechanism needs to be further investigated in the future.

For chronic inflammatory diseases or autoimmune diseases, extending drug half-life is an important strategy to improve patient compliance and reduce economic burden. Nbs have a short half-life in serum due to the lack of the Fc region of traditional antibodies. To overcome this deficiency, some studies have conjugated target molecules to albumin-targeting Nbs, thereby increasing the half-life of the target molecules [31–33]. This is because albumin prevents lysosomal degradation by binding to FcRn, which is highly expressed by many cells, resulting in a half-life of 17–19 days in humans [34–36]. In the current work, we fused a bivalent Nb targeting IL-5 with a Nb targeting albumin to form a new trivalent Nb. In primate studies, we found that this trivalent Nb achieved a half-life of 12 days, which is similar to the half-life of the IL-5-targeting mAb mepolizumab in monkeys [37].

In addition to half-life, which affects the frequency of dosing, an important consideration is the time the drug remains above the effective concentration in serum and tissue. Under the conditions of sufficient safety, traditional antibodies have difficulty achieving high-dose administration [38], making it difficult to further reduce the frequency of administration of antibodies with general antagonistic activity. Therefore, it is not difficult to understand that most of the approved traditional antibodies are administered once every 2–4 weeks. Compared to mAbs, bispecific antibodies are more potent in inhibiting cell function at the same dose. In this study, our trivalent Nb inhibited TF-1-cell proliferation with IC_50_ values ​​58-fold lower than those of mepolizumab. It is worth noting that in the primate study, the plasma concentration of this trivalent Nb was still above 100 ng/mL at 56 days, which was significantly higher than the 2 ng/mL concentration required to inhibit 90% of TF-1-cell proliferation. Consistently, 56 days after a single dose, we still observed a 73% and 53% decrease in peripheral blood eosinophil numbers from the baseline position in monkeys at the 2 mg/kg and 5 mg/kg doses, respectively. Therefore, our results support the administration of this trivalent Nb every 8 weeks, or even longer intervals, for the treatment of eosinophilic asthma.

Currently, most bispecific antibodies with extended half-lives have IgG-like asymmetric formats. Despite having a similar shape to native mAbs, their production typically requires at least two plasmids for the heterodimerized heavy chain and one for the common light chain, leading to greater production challenges [39]. Another difficulty is the correct assembly of bispecific antibody fragments. Several genetic and cellular engineering techniques, including quadroma technology, knobs-into-holes, common heavy chain, and CrossMab, have been developed to address this challenge, however, their yields from CHO cells are still low, mostly in the range of 0.03–1 g/L [40]. Therefore, the large-scale production of IgG-like asymmetric bispecific antibodies remains a major challenge. In contrast, our trivalent Nb consists of 3 VHHs and 3 linkers, which are cocloned into one plasmid. After expression in yeast, this trivalent Nb automatically formed a functional structure. Therefore, this design is very suitable for large-scale production, with a 1/5–1/10 cost advantage of conventional mAb. In fact, in our yeast expression system, the yield in a 100 L fermenter reached 7.5 g/L, which is much higher than that of the abovementioned IgG-like asymmetric bispecific antibody. Moreover, this trivalent Nb has good solubility and stability, showing excellent developability.

## Conclusions

In this work we reported a novel trivalent bispecific Nb targeting two different epitopes of IL-5 as well as HSA, showing potent IL-5/IL-5R blockage and TF-1-cell proliferation inhibitory activities. The trivalent IL-5-HSA Nb exhibited excellent pharmacokinetic properties and pharmacodynamic effects in primates, supporting 2–3 months dosing interval in future human studies. Moreover, the trivalent Nb could be produced on a large scale with high purity and good stability in a yeast system. Taken together, our study provides a novel long-acting anti-IL-5 trivalent Nb, with the potential to be a next-generation therapeutic agent for the treatment of eosinophilic asthma.

## Supplementary Information

**Additional file 1: Fig. S1.** Construction of the IL-5-specific Nbs library.

**Additional file 2: Fig. S2.** The biopanning results of the IL-5-specific Nbs library.

**Additional file 3: Fig. S3.** Original, full-length gel images.

**Additional file 4: Fig. S4.** The inhibitory effect of IL-5 single domain Nbs, homobivalent Nbs and heterobivalent Nb on TF-1-cell proliferation.

## Data Availability

All data generated or analyzed during this study are included in the article and additional file.

## Abbreviations

Nb: Nanobody
VHHs: Single-domain antibody fragments
mAb: Monoclonal antibody
ATCC: American Type Culture Collection
DMEM: Dulbecco’s Modified Eagle Medium
PBLs: Peripheral blood lymphocytes
CDR3: Complementary determining region 3
BLI: Biolayer interferometry
KD: Equilibrium dissociation constant
BSA: Bovine serum albumin
IC_50_: 50% Inhibitory concentration
EOS: Eosinophils
DO: Dissolved oxygen
SEC-HPLC: Size exclusion chromatography–high performance liquid chromatography
CEX-HPLC: Cation-exchange chromatography
CFU: Colony-forming units
FcRn: Neonatal Fc receptor
HSA: Human serum albumin
CSA: Monkey serum albumin
