# Supplementary material for: Preclinical development of a long-acting trivalent bispecific nanobody targeting IL-5 for the treatment of eosinophilic asthma
Source: Respir Res. 2022 Nov 19;23:316. doi: 10.1186/s12931-022-02240-1 (PMC9675287; doi:10.1186/s12931-022-02240-1)
Supplement: Supplementary file 1 — Additional file 1: Fig. S1. Construction of the IL-5-specific Nbs library. [file 12931_2022_2240_MOESM1_ESM.docx]

**Additional file 1**


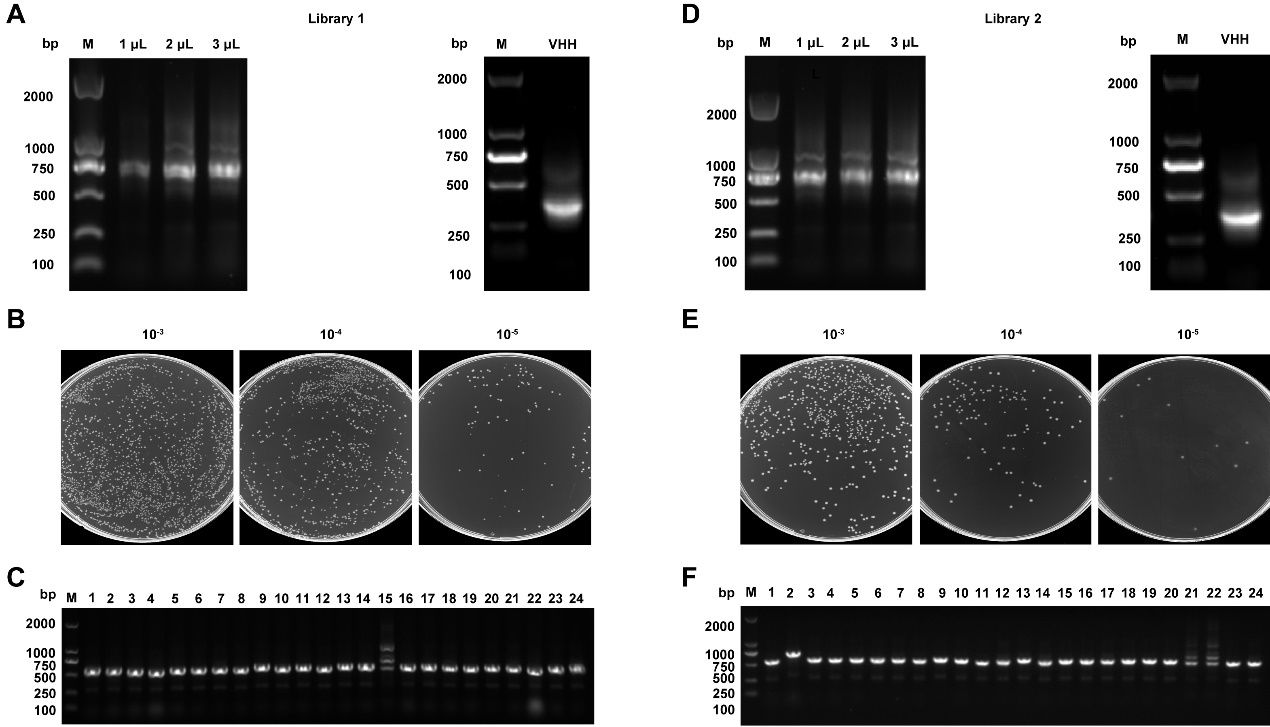


**Fig. S1** Construction of the IL-5-specific Nbs library. **A** The VHH gene fragments were amplified by a first PCR with an event band of 700 bp (left) and a second, nested PCR with an event band of 400 bp (right). **B** The capacity of library 1 was estimated by counting colony numbers. **C** The insertion rate of the library 1 was determined by performing PCR on randomly selected 24 colonies. **D-F** The VHH amplification (**D**), the capacity (**E**) and the insertion rate (**F**) of library 2. The cropping gels of nest-PCR products and library insertion rates were displayed.
