# Supplementary material for: Preclinical development of a long-acting trivalent bispecific nanobody targeting IL-5 for the treatment of eosinophilic asthma
Source: Respir Res. 2022 Nov 19;23:316. doi: 10.1186/s12931-022-02240-1 (PMC9675287; doi:10.1186/s12931-022-02240-1)
Supplement: Supplementary file 2 — Additional file 2: Fig. S2. The biopanning results of the IL-5-specific Nbs library. [file 12931_2022_2240_MOESM2_ESM.docx]

**Additional file 2**


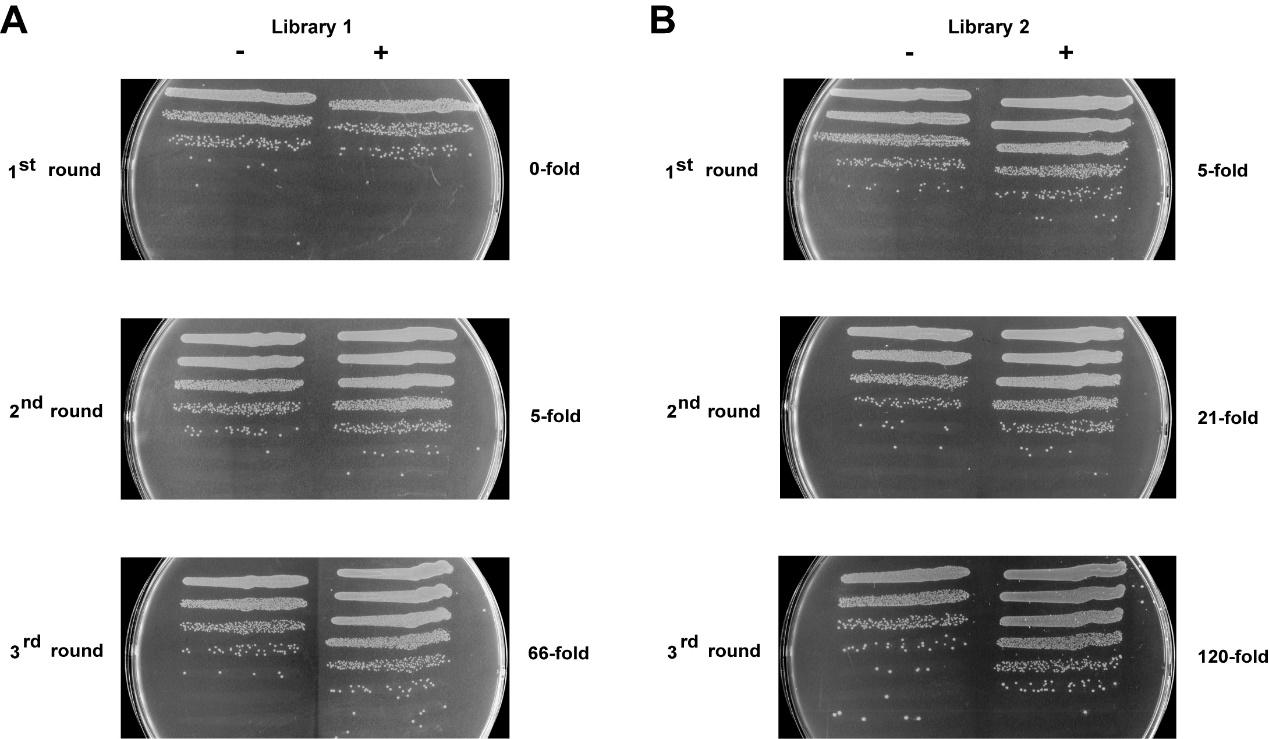


**Fig. S2** The biopanning results of the IL-5-specific Nbs library. The enrichment for phage particles of library 1(**A**) and library 2 (**B**) were detected after three consecutive rounds of panning. +: Phages transformed into TG1 cells after panning with IL-5-Fc. -: Phages panning with Fc were used as control.
