# Supplementary material for: Preclinical development of a long-acting trivalent bispecific nanobody targeting IL-5 for the treatment of eosinophilic asthma
Source: Respir Res. 2022 Nov 19;23:316. doi: 10.1186/s12931-022-02240-1 (PMC9675287; doi:10.1186/s12931-022-02240-1)
Supplement: Supplementary file 3 — Additional file 3: Fig. S3. Original, full-length gel images. [file 12931_2022_2240_MOESM3_ESM.docx]

**Additional file 3**


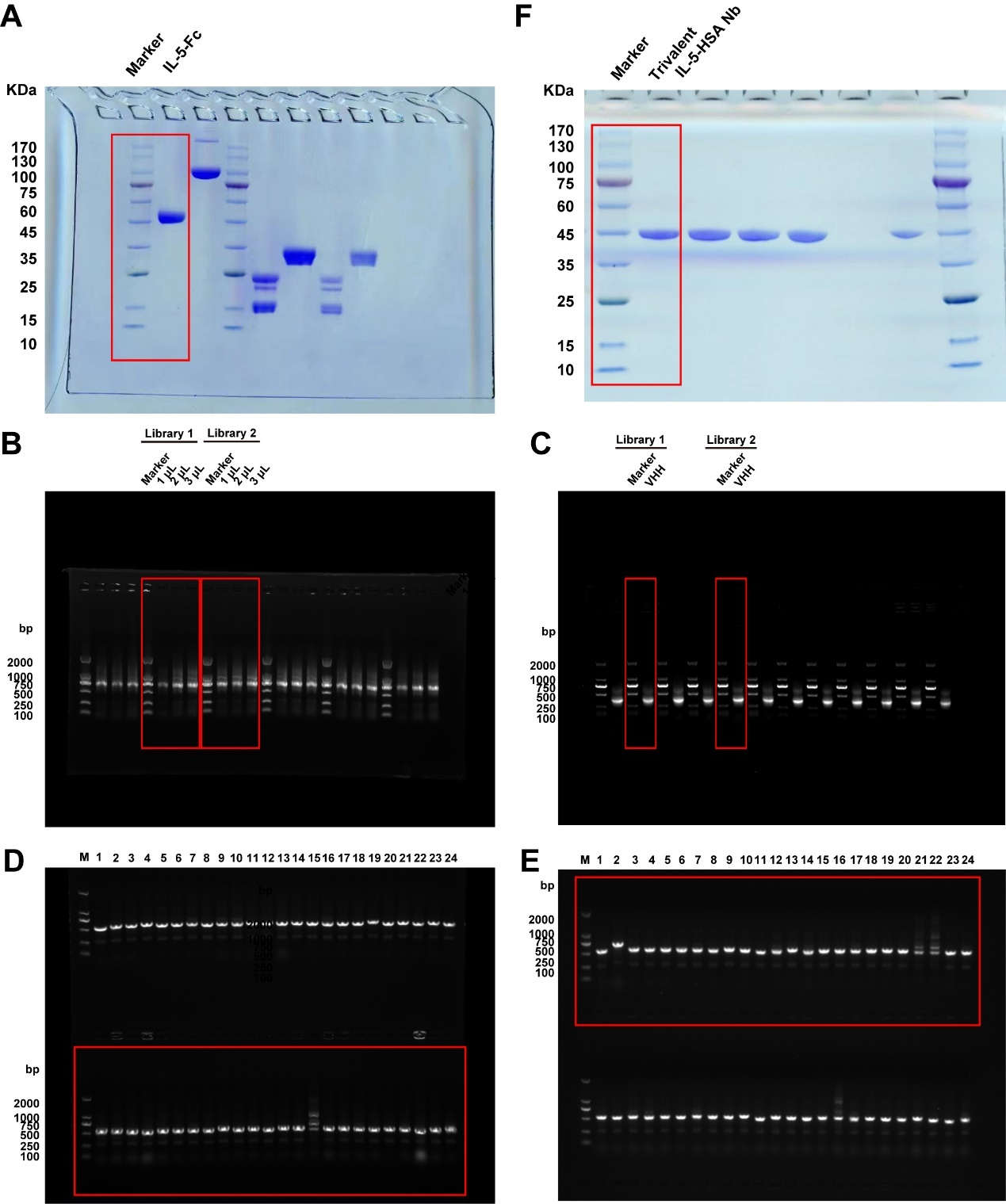


**Fig. S3** Original, full-length gel images. **(A)** The purified IL-5-Fc antigen **(B)** The amplified first PCR fragments from two libraries. **(C)** The amplified second PCR fragments from two libraries **(D)** The insertion rate of library 1. **(E)** The insertion rate of library 2. **(F)** The purified trivalent IL-5-HSA Nb. The cropped gel images displayed in Fig.1, Fig. 6 and Fig S1 were derived from the red rectangle labeled region of original images.
