# Supplementary material for: Preclinical development of a long-acting trivalent bispecific nanobody targeting IL-5 for the treatment of eosinophilic asthma
Source: Respir Res. 2022 Nov 19;23:316. doi: 10.1186/s12931-022-02240-1 (PMC9675287; doi:10.1186/s12931-022-02240-1)
Supplement: Supplementary file 4 — Additional file 4: Fig. S4. The inhibitory effect of IL-5 single domain Nbs, homobivalent Nbs and heterobivalent Nb on TF-1-cell proliferation. [file 12931_2022_2240_MOESM4_ESM.docx]

**Additional file 4**


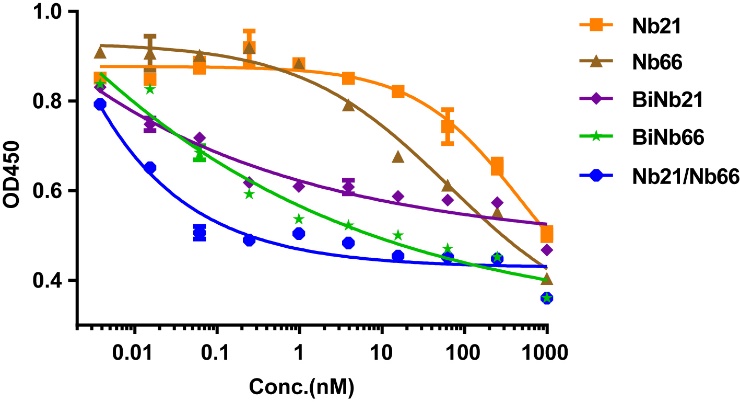


**Fig. S4** The inhibitory effect of IL-5 single domain Nbs, homobivalent Nbs and heterobivalent Nb on TF-1-cell proliferation. The inhibitory effect was determined by the CCK8 assay.
